# An Advanced Bio-Inspired Mantis Search Algorithm for Characterization of PV Panel and Global Optimization of Its Model Parameters

**DOI:** 10.3390/biomimetics8060490

**Published:** 2023-10-18

**Authors:** Ghareeb Moustafa, Hashim Alnami, Sultan Hassan Hakmi, Ahmed Ginidi, Abdullah M. Shaheen, Fahad A. Al-Mufadi

**Affiliations:** 1 Electrical Engineering Department, Jazan University, Jazan 45142, Saudi Arabia; halnami@jazanu.edu.sa (H.A.); shhakmi@jazanu.edu.sa (S.H.H.); 2 Electrical Engineering Department, Faculty of Engineering, Suez University, Suez 43533, Egypt; ahmed.ginidi@eng.suezuni.edu.eg (A.G.); abdullah.mohamed.eng19@suezuni.edu.eg (A.M.S.); 3 Mechanical Engineering Department, College of Engineering, Qassim University, Buraydah 51452, Saudi Arabia; almufadi@hotmail.com

**Keywords:** Mantis Search Algorithm, PV panel characterisation, PV model parameters optimisation, root mean square error minimisation

## Abstract

Correct modelling and estimation of solar cell characteristics are crucial for effective performance simulations of PV panels, necessitating the development of creative approaches to improve solar energy conversion. When handling this complex problem, traditional optimisation algorithms have significant disadvantages, including a predisposition to get trapped in certain local optima. This paper develops the Mantis Search Algorithm (MSA), which draws inspiration from the unique foraging behaviours and sexual cannibalism of praying mantises. The suggested MSA includes three stages of optimisation: prey pursuit, prey assault, and sexual cannibalism. It is created for the R.TC France PV cell and the Ultra 85-P PV panel related to Shell PowerMax for calculating PV parameters and examining six case studies utilising the one-diode model (1DM), two-diode model (1DM), and three-diode model (3DM). Its performance is assessed in contrast to recently developed optimisers of the neural network optimisation algorithm (NNA), dwarf mongoose optimisation (DMO), and zebra optimisation algorithm (ZOA). In light of the adopted MSA approach, simulation findings improve the electrical characteristics of solar power systems. The developed MSA methodology improves the 1DM, 2DM, and 3DM by 12.4%, 44.05%, and 48.88%, 28.96%, 43.19%, and 55.81%, 37.71%, 32.71%, and 60.13% relative to the DMO, NNA, and ZOA approaches, respectively. For the Ultra 85-P PV panel, the designed MSA technique achieves improvements for the 1DM, 2DM, and 3DM of 62.05%, 67.14%, and 84.25%, 49.05%, 53.57%, and 74.95%, 37.03%, 37.4%, and 59.57% compared to the DMO, NNA, and ZOA techniques, respectively.

## 1. Introduction

### 1.1. Motivation and Incitement

The utilisation of renewable energy resources is gaining prominence as a result of growing fuel costs and global warming. Due to its low maintenance requirements, environmental friendliness, and widespread availability, solar energy has become progressively more popular as a steady and trustworthy source of clean energy in recent years.

Solar energy is used for numerous purposes other than just producing electricity, including water heating, farming, household appliances, automobile battery charging, lighting, rooftops, and agriculture. However, operations for large-scale power generation have been significantly hampered by the unpredictable nature of solar energy. To enhance dynamic energy management and grid operations in emergency situations, simulation investigations using accurate models of solar PV systems must be conducted [1]. A mathematical model with real-time voltages and currents and accurate parameters can produce accurate results and boost system performance.

Researchers have examined a variety of techniques in recent years to precisely estimate the PV model’s characteristics. These techniques can be categorised into three main groups: analytical approaches, deterministic approaches, and meta-heuristic approaches [2]. The foundation of analytical approaches is the analysis of mathematical equations in accordance with the features of the issues. Hence, these techniques are simple to use but may diminish the accuracy of the solution because of the value of arbitrarily specified points and the required hypotheses [3].

### 1.2. Literature Review

The deterministic approaches, in contrast to the analytical technique, typically use gradient-based techniques, such as the Lambert W-functions [4] and Newton–Raphson [5], which are susceptible to starting values and easily caught in local optima. In recent years, a variety of meta-heuristic methods have been used to resolve PV model parameter estimate issues in order to get around these shortcomings [6], such as the improved shuffled complex evolution algorithm (ISCE) [7], the fireworks algorithm (FA) [8], the Ant Lion Optimizer (ALO) [9], the artificial bee colony (ABC) [10], the flower pollination algorithm (FPA) [11], the chaotic whale optimisation algorithm (CWO) [12], and JAYA [13]. Even though many meta-heuristic approaches have produced results that are generally adequate, there is still a lot of opportunity for improvement in terms of the reliability, accuracy, convergence speed, and complexity of tuning parameters for competitive meta-heuristic algorithms to solve PV parameter estimation.

The solar model parameters have been determined using the enhanced Harris–Hawk algorithm (CCNMHHO) by Liu et al. [14]. The moth flame method (MFO) has been applied to the parameter identification of PV modules by Zhang et al. [15]. The additional mechanism improved local mining capabilities and global convergence, resulting in exceptional performance in the 1DM and 2DM PV models. An enhanced augmented mutation HHO has been suggested by Ridha et al. [16], in order to generate a model that is more effective and stable and to accurately determine the parameters of the PV system. The algorithm’s convergence can be accelerated by the suggested approach. In [17], the adversarial-based exploratory technique with the chaotic drift mechanism has been merged into HHO to enable them to appropriately analyse the solar cell simulation parameters of the 1DM, 2DM, and solar models of PV modules. Additionally, they confirmed the technique’s efficacy in identifying crucial factors under various lighting and temperature conditions. The improved Ant Lion Optimizer (IALO), suggested by Wu et al. [18], has been introduced for parameter evaluation. IALO had successful outcomes with the photovoltaic model. In order to assess the unknown parameters for 1DM and 2DM, Chen et al. [19] suggested an improved sine–cosine technique called ISCA. Even though nature-inspired metaheuristics and their variations outperform deterministic methods in terms of solution quality and speed, they have a number of disadvantages. For instance, the technique’s convergence speed could be improved. In addition, the method is somewhat specialised, and its high performance is only limited to specific types of optimisation problems, which restricts its application areas. For the purpose of finding the unknown parameters of various solar models and optimising the optimal parameters of photovoltaic models in various situations, Merchaoui et al. [20], ref. [21] suggested an adaptive variational particle swarm optimisation (PSO) algorithm. In order to choose the best design options, Ridha et al. [22] offered a thorough review based on multi-objective optimisation and multi-criteria methodologies for stand-alone PV system design. In [23], the evaluation of the characteristics of solar cells and PV modules has been determined using orthogonal learning (OL) and generalised opposition-based learning (GOBL) techniques. An enhanced technique based on the salp swarm algorithm has been proposed by Abbassi et al. [24], which applies an opposition-based learning approach to the parameter recognition issue associated with solar cells. In [25], the Artificial Humming Bird Optimisation (AHBO) algorithm was proposed with three objective functions, which are the root mean square error (RMSE), the Lambert W function, and the iterate Newton–Raphson approach for the 1DM and 2DM models.

In many areas of research, the optimisation problem involves a single issue that has more than one possible solution. Therefore, its goal is to identify the best choice from all of the feasible options. The optimisation problems are generally divided into three parts: the decision variables, constraints, and objective [26]. In this computational field, problem-solving algorithms have been defined as deterministic or stochastic [27]. Stochastic approaches tackle optimisation problems by investigating the resulting searching space at random and applying arbitrary operators. Such approaches generate a group of viable solutions to a certain problem before repeatedly enhancing them in order to arrive at a suitable one [28,29]. In [30], an innovative metaheuristic algorithm called the Black Widow Optimisation Algorithm (BWOA), which is inspired by the hunting behaviour of black widow spiders, was designed for the parameter extraction of PV cells and panels. In this study, two PV cells of amorphous silicon (aSi) and RTC France and two PV panels of PVM 752 GaAs and PWP201 were considered. It highlighted the effectiveness of the BWOA in accurately determining the parameters, but this study was limited to the simplified 1DM and 2DM only.

The optimal response to an optimisation problem is the global optimum. However, there is no guarantee that the methods utilised will yield such an ideal result. Thus, the solution generated by an optimiser for any particular issue is known as a quasi-optimal [31]. Metaheuristic strategies require being capable of executing and overseeing searches at the global, as well as local levels, in order to organise a successful investigation in the problem-solving domain. Globally, exploration contributes to an in-depth evaluation in the area of problem solution, directing the focus away from the best local areas [32,33].

### 1.3. Contribution and Paper Organisation

Correct modelling and estimation of solar cell characteristics are crucial for effective performance simulations of PV panels, necessitating the development of creative approaches to improve solar energy conversion. The investigated problem of the characterisation of PV panel aims at finding the unknown model parameters regarding the electrical equivalent circuits of PV systems. Three different models are usually generated which are 1DM, 2DM, and 3DM. In this study, the Mantis Search Algorithm (MSA) is presented for the PV parameter extraction issue. The proposed MSA is a unique nature-inspired metaheuristic optimisation algorithm developed in [34], which uses the hunting and sexual cannibalism behaviour of praying mantises. In order to further enhance the exploration and exploitation operators, the newly developed MSA uses three optimisation operators: searching for prey, attacking prey, and sexual cannibalism. Compared to several metaheuristic algorithms, the proposed MSA can escape from the local optima and is easy to implement. Moreover, it maintains population diversity throughout the optimisation process, and it possesses a high capacity for balancing operators engaged in exploration and extraction. Furthermore, it is able to solve the unimodal test functions because of its powerful exploitation operator. The MSA is evaluated to analyse the parameter estimation of photovoltaic modules. The outcomes illustrate the MSA’s ability to address the estimation of photovoltaic modules with high efficiency.

The following are the primary contributions to this paper:

The MSA bio-inspired optimisation is established for the first time in the present research, in accordance with the author’s knowledge, for properly obtaining the electrical PV parameters.

The original MSA, including the pursuit of prey, attack prey, and sexual cannibalism, is designed for estimating PV parameters and employed for two commercial PV systems of RTC France PV cell and the Ultra 85-P PV panel.

Its usefulness is proven considering the 1DM, 2DM, and 3DM, by comparing it to recent optimisation techniques, such as the neural network optimisation algorithm (NNA) [35], dwarf mongoose optimisation (DMO) [36], and the zebra optimisation algorithm (ZOA) [37]. Additionally, the proposed MSA approach achieves effective superiority and consistency when contrasted with other previously reported results.

This research is divided into five parts: Section 2 provides a mathematical explanation of the 1DM, 2DM, and 3DM systems, whereas Section 3 demonstrates the designed MSA process. In addition, Section 4 illustrates a detailed explanation of the obtained simulation results via MSA, NNA, DMO, ZOA, and numerous documented approaches. Section 5 contains the paper’s concluding notes.

## 2. Problem Formulation of Solar PV Parameters Extraction

This part covers the computational modelling of photovoltaic panels, including 1DM, 2DM, and 3DM systems. A description of the objective function is then used to tackle the parameter selection problem for the previously discussed PV systems [38].

### 2.1. PV Equivalent Circuit-Based on 3DM

**Figure 1:**
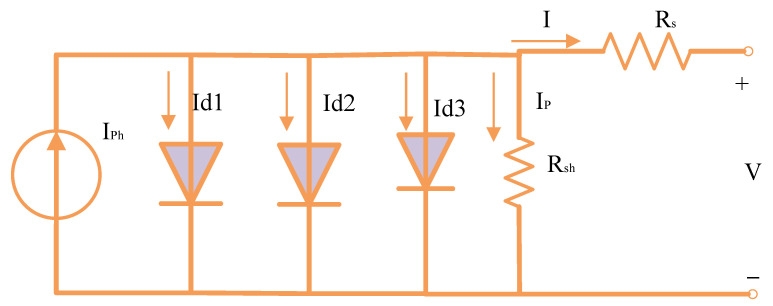
Representation of the 3DM circuit.

The PV Equivalent circuit-based on 3DM is depicted in Figure 1 [39].

The shunt resistance current (*I_p_*) sign, photocurrent (*I_ph_*), diode current (*I_d_*), and output current (*I*) are capable of being stated to be produced in the equation that follows [40]:

(1) $$I=I_{Ph}-I_{p}-I_{d1}-I_{d2}-I_{d3}$$

The shunt resistance current, *I_p_*, might be calculated using the formula as follows:

(2) $$I_{p}=\frac{IR_{s}+V}{R_{sh}}$$

Equation (3) develops the link between the output voltage, output current, and other different factors within the 3DM through the use of the above equations.

(3) $$I=I_{Ph}-I_{s1}\left[\exp\left(\frac{IR_{s}+V}{\eta_{1}V_{thr}}\right)-1\right]-I_{s2}\left[\exp\left(\frac{IR_{s}+V}{\eta_{2}V_{thr}}\right)-1\right]-I_{s3}\left[\exp\left(\frac{IR_{s}+V}{\eta_{3}V_{thr}}\right)-1\right]-\frac{V}{R_{sh}}-I\frac{R_{s}}{R_{sh}}$$

where $\eta_{1}$, $\eta_{2}$, and $\eta_{3}$ denote the ideality parameters of the diodes; *I_s_*_1_, *I_s_*_2_, and *I_s_*_3_ denote the 3D reverse saturation currents; and *V_thr_* denotes the junction thermal voltage as specified in Equation (4).

(4) $$V_{thr}=\frac{K_{B}\times T}{q_{c}}$$

where *K_B_* stands for Boltzmann’s constant, *q_c_* stands for the electron charge, and *T* stands for temperature in kelvin.

Equation (4) shows that there are nine variables (*I_s_*_1_, *I_s_*_2_, *I_s_*_3_, *I_ph_*, *R_sh_*, *R_s_*, $\eta_{1}$, $\eta_{2}$, and $\eta_{3}$) needed for extracting the 3DM [41].

### 2.2. PV Equivalent Circuit-Based on 2DM

**Figure 2:**
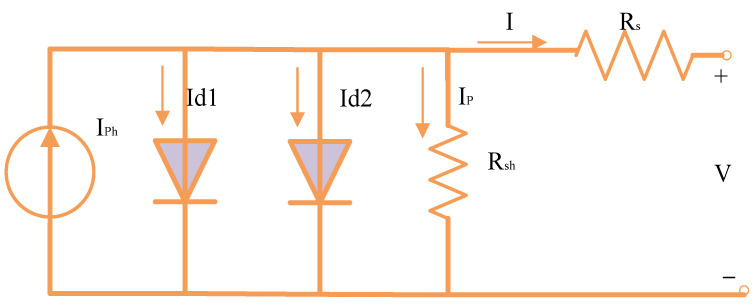
Representation of the 2DM circuit.

The 2DM is developed by simplifying the 3DM through deleting the third diode-branch, as shown in Figure 2 [42].

In accordance with this reduced form, Equation (5) develops the link between the output voltage, output current, and other different factors in the 2DM through the use of the preceding equations.

(5) $$I=I_{ph}-I_{s1}\left[\exp\left(\frac{IR_{s}+V}{\eta_{1}V_{thr}}\right)-1\right]-I_{s2}\left[\exp\left(\frac{IR_{s}+V}{\eta_{2}V_{thr}}\right)-1\right]-\frac{V}{R_{sh}}-I\frac{R_{s}}{R_{sh}}$$

Equation (5) shows that a total of seven parameters (*I_s_*_1_, *I_s_*_2_, *I_ph_*, *R_sh_*, *R_s_*, $\eta_{1}$, and $\eta_{2}$) needed for extracting for 2DM.

### 2.3. PV Equivalent Circuit-Based on 1DM

**Figure 3:**
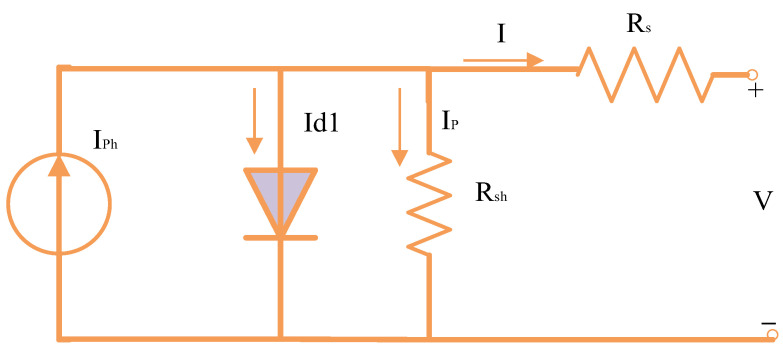
Representation of 1DM circuit.

As shown in Figure 3, the 1DM is created by simplifying the 2DM by removing the second diode-branch. As a result, Equation (6) develops the following link between the output current and other different parameters [43]:

(6) $$I=I_{Ph}-I_{s1}\left[\exp\left(\frac{IR_{s}+V}{\eta_{1}V_{thr}}\right)-1\right]-\frac{V}{R_{sh}}-I\frac{R_{s}}{R_{sh}}$$

Equation (6) shows that a total of five parameters (*I_s_*_1_, *I_ph_*, *R_sh_*, *R_s_*, and $\eta_{1}$) needed for extracting for 1DM.

### 2.4. Objective Model

Before developing goal functions which are suitable for various computations, it becomes crucial to test the output voltage and output current for every model. Consequently, the function’s goal is to determine the variance between the experimental and calculated currents within the constructed model [44]. Therefore, the minimisation of the RMSE is modelled as follows:

(7) $$RMSE=\sqrt{\frac{1}{P\times N}\left(\sum_{K=1}^{PN}{\left(I_{cal}^{K}(V_{\exp}^{K},x)-I_{\exp}^{K}\right)}^{2}\right)}$$

where *V_exp_^K^* and *I_exp_^K^* are the measured voltage and current, respectively, and *PN* is the total amount of measured points of data. In addition, the symbol (*x*) shows the PV identifying variable issue, which is concerned with finding a solution that minimises the RMSE function.

## 3. Developing MSA for Best Extraction of PV Parameters

The three basic stages of MSA are mathematically represented in this section. The location of mantises denotes population initialisation in the first step. The second step reflects the exploration phase, the third the exploitation phase, and the fourth the discussion of sexual cannibalism.

**Figure 4:**
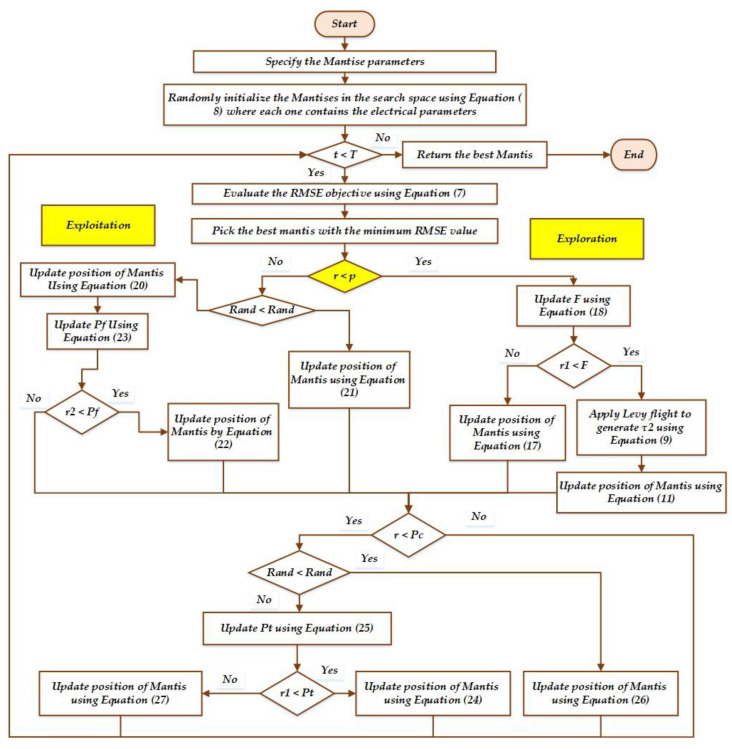
Main procedures of MSA.

Before initiating the optimisation procedure, the following parameters are set to the MSA; these parameters are *T* is maximum iteration; *A* is length of an archive, *N* is population size, *p* is probability to exchange between the exploitation and exploration stages, *a* is strike’s failure probability, *ρ* is gravitational acceleration rate of the mantis’s strike, *P* is the recycling factor to trade off between spearers and pursuers, and *P_c_* is used to estimate the sexual cannibalism percentage. Finally, the flowchart of MSA is given in Figure 4.

### 3.1. Initial Population

Every mantis in MSA represents a potential solution to an optimisation problem. A two-dimensional matrix *x* of size *N* solutions (mantises) × *D* (dimensional search space) can be created. Furthermore, as described in Equation (1), a vector containing an arbitrary initialisation within the lower and upper borders of the optimisation problem can be used:

(8) $${\vec{x}}_{i}^{t}={\vec{x}}_{}^{l}+\vec{r}\times ({\vec{x}}_{}^{u}-{\vec{x}}_{}^{l}),$$

where ${\vec{x}}_{i}^{t}$ is the location of each Mantis *i* at function assessment *t*; ${\vec{x}}_{}^{l}$, and ${\vec{x}}_{}^{u}\;$ illustrate the lower and upper boundaries for the *j*-dimension, respectively; and $\vec{r}$ is a vector that contains numbers randomly generated between 0 and 1, in accordance with the uniform distribution.

### 3.2. Exploration Stage

In MSA, Lévy flight and the normal distribution are integrated to cover both short and large step sizes that represents the seeking of the MSA predators to look for prey away from their hiding places. Lévy flights are, in general, random walks whose step length is derived from the Lévy distribution, generally in terms of a simple power-law formula $L\left(x\right)∼{\left|x\right|}^{-1-\beta }$ where 0<β≤2 is an index. A simplified form of the Lévy distribution can be expressed mathematically as:

(9) $$L(x,\gamma ,\phi )=\left\{\begin{array}{l} \begin{array}{cc} \sqrt{\frac{\gamma }{2\pi }}\exp(-\gamma /(2x-2\varphi ))\frac{1}{{\left(x-\varphi \right)}^{1.5}} & if\;0<\varphi <x<\infty \\ 0 & if\;x<0 \end{array} \end{array}\right.$$

where ϕ > 0 is a minimum step and γ  is a scale parameter. As γ →∞, the model is clearly altered to:

(10) $$L(x,\gamma ,\phi )=\frac{1}{x^{1.5}}\sqrt{\frac{\gamma }{2\pi }}$$

This is a specific case of the generalised Lévy distribution.

This hybridisation between the normal distribution and Lévy flight allows to simulate the actions of pursuers as they look for their prey as follows:

(11) $${\vec{x}}_{i}^{t+1}=\left\{\begin{array}{ll} {\vec{x}}_{i}^{t}+\vec{\tau_{1}}\times ({\vec{x}}_{i}^{t}-{\vec{x}}_{a}^{t})+\left|\tau_{2}\right|\times \vec{U}\times ({\vec{x}}_{a}^{t}-{\vec{x}}_{b}^{t}), & if\;r_{1}\leq r_{2}\\ {\vec{x}}_{i}^{t}\times \vec{U}+({\vec{x}}_{a}^{t}+\vec{r_{3}}\times ({\vec{x}}_{b}^{t}-{\vec{x}}_{c}^{t}))\times (1-\vec{U}), & otherwise \end{array}\right.$$

where ${\vec{x}}_{i}^{t+1}\;$ and ${\vec{x}}_{i}^{t}$ denote, respectively, the location of *i*^th^ solution (mantis) at function evaluation *t +* 1 and *t*. Moreover, the symbol $\left|\tau_{2}\right|$ represents a random number using the normal distribution with a mean of 0 and a standard deviation of 1, while the symbol $\vec{\tau_{1}}$ manifests a numerical vector created using the Lévy flight approach. Furthermore, $r_{1}$ and $r_{2}$ illustrate numbers randomly created using the uniform distribution between 0 and 1, while $\vec{r_{3}}$ is a vector including numerical values randomly created using the uniform distribution in the range (0, 1). ${\vec{x}}_{a}^{t}$, ${\vec{x}}_{b}^{t}$, and ${\vec{x}}_{c}^{t}$ manifest solutions drawn randomly from the existing population, such that ${\vec{x}}_{a}^{t}\neq {\vec{x}}_{b}^{t}\neq {\vec{x}}_{c}^{t}\neq {\vec{x}}_{i}^{t}$, whereas $\vec{U}$ represents a binary vector created using the following formula:

(12) $$\vec{U}=\left\{\begin{array}{l} 0\;\;\vec{r_{4}}<\vec{r_{5}}\\ 1\;\;otherwise \end{array}\right.,$$

where $\vec{r_{4}\;}$ and $\vec{r_{5}\;}$ manifest a vector including numerical values randomly created using the uniform distribution in the range (0, 1). Each *j*^th^ dimension of the two mentioned vectors in are compared to each other, and consequently, the *j*^th^ element in the binary vector $\vec{U}$ will be set to 0, if the prior vector has a smaller value, otherwise, it will be set to 1.

While remaining on the ground or concealed in the woods, the ambuscade mantis waits for prey to approach within striking distance. The following formula can be used to simulate this behaviour mathematically:

(13) $${\vec{x}}_{i}^{t+1}={\vec{x}}_{i}^{t}+\alpha \times ({\vec{x}}_{ar}^{t}-{\vec{x}}_{a}^{t})$$

where *α* is a variable that regulates the mantis’ head position to allow it to cover the ambuscade distance, and ${\vec{x}}_{ar}^{t}$ is a solution vector representing the *i*^th^ mantis position, which is picked from the archive randomly. The variable *α* can be formulated mathematically, as depicted in the following equation:

(14) $$\alpha =\cos(\pi r_{6})\times \mu$$

where $r_{6}$ illustrates number randomly created using the uniform distribution between 0 and 1, and *μ* represents a distance factor that can be calculated as depicted in the next equation:

(15) $$\mu =\left(1-\frac{t}{T}\right)$$

where *T* manifests the highest number of function assessments.

The prey may come into the mantis’s range of attack as it moves quickly around the surrounding areas in search of food. The behaviour of getting the prey to the ambuscade distance can be calculated using the following equation:

(16) $${\vec{x}}_{i}^{t+1}={\vec{x}}_{ar}^{t}+(2\times r_{7}-1)\times \mu \times ({\vec{x}}_{}^{l}+\vec{r_{8}}\times ({\vec{x}}_{}^{u}-{\vec{x}}_{}^{l}))$$

where $r_{7}$ illustrate numbers randomly created using the uniform distribution between 0 and 1; $\vec{r_{8}}$ is a vector includes numerical values randomly created using the uniform distribution in the range (0, 1).

The distance between the invisible locations and the prey is long, as depicted in Equation (7) at the start of the optimisation process. This distance steadily decreases when the current iteration is increased, because the prey is transferred in the mantis’s direction. The following mathematical formulation describes how mantises and their prey engage in ambuscade behaviour:

(17) $${\vec{x}}_{i}^{t+1}=\left\{\begin{array}{cc} {\vec{x}}_{i}^{t}+\alpha \times ({\vec{x}}_{ar}^{t}-{\vec{x}}_{a}^{t}), & r_{9}\leq r_{10}\\ {\vec{x}}_{ar}^{t}+(2r_{7}-1)\times \mu \times ({\vec{x}}_{}^{l}+\vec{r_{8}}\times ({\vec{x}}_{}^{u}-{\vec{x}}_{}^{l})), & otherwise \end{array}\right.$$

where $r_{9}$ and $r_{10}$ illustrate numbers randomly created using the uniform distribution between 0 and 1 to accomplish a trade-off in behaviour between mantises’ ambuscade and prey.

Then, utilising the recycling control factor, which separates the optimisation process into portions and aids in examining the potential search space of an optimisation issue, the behaviours of pursuers and spearers are incorporated into the suggested optimiser. This factor is expressed numerically, as depicted in the next equation:

(18) $$F=1-\frac{t\%(T/P)}{T/P}$$

where the symbol ‘%’ represents the modulus remainder operator and *P* represents an integer that expresses the number of cycles that are utilised to create an exchange between Equations (11) and (17).

### 3.3. Attacking the Prey: Exploitation Stage

When the target is too close, the mantis terminates its hunt by attacking it. It is thought that the mantis can sense when it is appropriate to attack its prey. The mantis attacks its prey using its front legs. The magnitude of the mantis strikes velocity when attacking prey is calculated using the sigmoid curve with a constant value. The following equation can be used to mathematically calculate the magnitude of a mantis’s front legs’ striking velocity ($v_{s}$) in the direction of its prey:

(19) $$v_{s}=\frac{1}{1+e^{l\rho }}$$

where *ρ* is a constant value evaluated in the succeeding experiments and denotes the gravitational acceleration rate of the mantis’s strike. To manage the gravitational acceleration rate, the symbol (*l*) is a number created between −1 and −2, and any value of l that is close to −1 and −2 minimises and maximises the striking velocity magnitude close to 0 and 1, respectively. The mantis recognises that it is not the right time to attack the prey when it $v_{s}\;$ reaches a value of 0, but when it approaches a value of 1, it moves fast to attack the target prey and eats it before it can flee. The formula below updates each mantis’ behaviour when grabbing its prey:

(20) $$x_{i,j}^{t+1}=(x_{i,j}^{t}+x_{j}^{*})/2.0+v_{s}\times (x_{j}^{*}-x_{i,j}^{t}),$$

where $x_{j}^{*}$ denotes the current location for the best solution obtained; $x_{i,j}^{t+1}$ signifies the new position at function evaluation *t* + 1, for the mantis *j*^th^ dimension of *i*; and $x_{i,j}^{t}$ denotes the prey’s location to minimise the distance between them and to accelerate the attacking process.

The mantis must occasionally reverse its trajectory after a failed strike to succeed. where the mantis modifies its direction in accordance with the movement of two mantises chosen at random from the population, according to the formula shown in Equation (21).

(21) $$x_{i,j}^{t+1}=x_{i,j}^{t}+r_{12}\times (x_{a,j}^{t}-x_{b,j}^{t}),$$

where the symbols ($x_{a}^{t}$ and $x_{b}^{t}$) deonte two mantises randomly chosen to decide the current mantis direction before striking again, while $r_{12}$ illustrate numbers randomly created using the uniform distribution between 0 and 1.

If the mantis strike fails, the local optima has captured it. In order to leave the local optima, individuals need to have strong exploitation and exploration skills. The algorithm is updated so that the mantises might adopt better locations for striking their prey once more in the following mathematical model, which prevents the algorithm from entering the local optima.

(22) $$x_{i,j}^{t+1}=x_{i,j}^{t}+e^{2l}\times \cos(2l\pi )\times \left|x_{i,j}^{t}-{\vec{x}}_{ar,j}^{l}\right|+(2r_{13}-1)\times (x_{j}^{u}-x_{j}^{l})$$

where $r_{13}$ illustrate numbers randomly created using the uniform distribution between 0 and 1. Equation (14) is employed with a failure probability in the suggested algorithm for the sake of preventing becoming stuck in local minima and to speed up convergence to the best solution.

The probability formulated in Equation (23) drops gradually with increased current function assessment, to reduce the exploration process and gradually increase the exploitation operator to speed up convergence to the near-optimal solution.

(23) $$P_{f}=a\times (1-\frac{t}{T})$$

where *a* is a pre-determined value that regulates exploration and exploitation operations and ranges from 0 to 1. A low value for this parameter decreases exploration while increasing exploitation. Within MSA, Equations (20) and (21) are randomly switched, and Equation (22) is applied following Equation (20), in accordance with the probability covered in Equation (20). $r_{4}$ is a number created at random for each dimension in the updated solution, and it is constant for all dimensions in each solution, while $r_{2\;}$ is a random number in the range [0, 1] for each solution.

### 3.4. Sexual Cannibalism

Sexual cannibalism occurs in praying mantises when the females consume the males either during or right after copulation. Female praying mantises lure males to their positions as the first action in this behaviour, which is mathematically recreated in line with the following formula:

(24) $${\vec{x}}_{i}^{t+1}={\vec{x}}_{i}^{t}+{\vec{r}}_{16}\times ({\vec{x}}_{i}^{t}-{\vec{x}}_{a}^{t}),$$

where ${\vec{x}}_{i}^{t}$ signifies the praying mantises female, ${\vec{x}}_{a}^{t}$ manifests a solution that is chosen from the population randomly to characterise the male drawn using the female for reproducing and it is being eaten, and ${\vec{r}}_{16}$ manifests a vector including numerical values randomly created using the uniform distribution in the range (0, 1) to stand for the attraction variable.

In contrast to virgin females, who are more likely to draw male attention, mated females only sometimes attract males. Typically, this probability, designated *P_t_*, is expressed mathematically as follows:

(25) $$P_{t}=r_{17}\times \mu$$

where *P_t_* means the probability of mating between the females and males, $r_{17}$ illustrates a number randomly created using the uniform distribution between 0 and 1.

The male then mates with the female using the genetic operators’ uniform crossover operator to create a new offspring, as expressed in the following equation:

(26) $${\vec{x}}_{i}^{t+1}={\vec{x}}_{i}^{t}\times \vec{U}+(x_{11}^{t}+{\vec{r}}_{18}^{}\times (-{\vec{x}}_{11}^{t}+{\vec{x}}_{i}^{t}))\times (1-\vec{U}),$$

where ${\vec{r}}_{18}$ represents a vector including numerical values randomly created using the uniform distribution in the range (0, 1); and $x_{11}^{t}$ signifies the value of the *l*^th^ dimension. The following mathematical equation can represent the female that eats the male during or after mating:

(27) $${\vec{x}}_{i}^{t+1}={\vec{x}}_{a}^{t}\times \cos(2\pi l)\times \mu ,$$

where ${\vec{x}}_{a}^{t}$ represents the male, and the usage of the term (cos (2π*l*)) is to allow the female flexibility, during the eating process, to turn the male around, and *μ* is the eaten part from the male.

In the MSA, the search mechanisms are designed to balance exploration and exploitation in order to efficiently search the solution space. By combining these search mechanisms, MSA aims to achieve a balance between exploration and exploitation. The random search and global search mechanisms promote exploration by diversifying the population and exploring new regions, while the local search mechanism focuses on exploitation by refining promising solutions. The adaptive search mechanism dynamically adjusts the balance between exploration and exploitation based on the algorithm’s performance, ensuring an efficient search process that can effectively explore and exploit the solution space. From the flowchart, the yellow diamond indicates the adaptive control between exploration and exploitation characteristics where A probability (p) of 50% is utilised to exchange between the exploration and exploitation stages.

## 4. Simulation Results

This part studies the RTC France PV cell and the Ultra 85-P PV panel employing the proposed MSA technique. The primary test investigation uses a commercially available silicon solar RTC France cell that works at 33 degrees Celsius and exhibits a sun radiance of 1000 W/m^2^. It has a short-circuited current, open circuit voltage, maximum point voltage, and current of 0.7605 A, 0.5727 V, 0.4590 V, and 0.6755 A, respectively. Another test case is utilised for additional practical validations of the proposed MSA technique in obtaining 1DM, 2DM, and 3DM models. The commercialised module Ultra 85-P from the Shell PowerMax manufacturer is considered. This panel has a maximum output of 85 W at STC with a tolerance of 5% and is made up of 36 monocrystal-line PV cells linked in series. This module possesses a fill factor of 70.3% and an efficiency of 13.4%. This panel is 120.0 cm in length, 52.70 cm in width, 3.40 cm in depth, weighs 7.5 kg, and is protected by a 20 A series fuse. This module’s complete datasheet is available in [45].

Table 1 shows the upper and lower limits for the unknown electrical parameters for these two PV systems. The MSA method has been studied and employed to address parameter extraction concerns using the 1DM, 2DM, and 3DM systems, in contrast to recently developed optimisers of neural network optimisation algorithm (NNA) [35], dwarf mogoose optimisation (DMO) [36,46,47], and zebra optimisation algorithm (ZOA) [37,48]. Each of the examined computations, MSA, NNA, DMO, and ZOA, are run with an identical number of iterations (1000) and solutions (100). The settings of the MSA are stated in Table A1 in the Appendix A.

### 4.1. First Test Investigation: RTC France Cell

#### 4.1.1. Case 1: Application for 1DM System

The MSA, NNA, DMO, and ZOA techniques are employed to decrease the RMSE objective function for the 1DM model of a commercial RTC France silicon PV cell. Table 2 displays the best outcomes for each algorithm for the five 1DM parameters that were unknown during the experiment. The results demonstrate that MSA has excellent performance in contrast to its peers, which are newly developed techniques, such as NNA, DMO, and ZOA. The table demonstrates that MSA earned the best RMSE value of 9.9869 × 10^−4^ from the comparison algorithms, which achieve RMSE values of 10.212 × 10^−4^ and 10.309 × 10^−4^ for DMO and ZOA, respectively. Moreover, excellent improvements, according to these findings, have been obtained using the proposed MSA of 3.45%, 0.09%, and 4.35% with regard to the DMO, NNA, and ZOA techniques, respectively.

**Figure 5:**
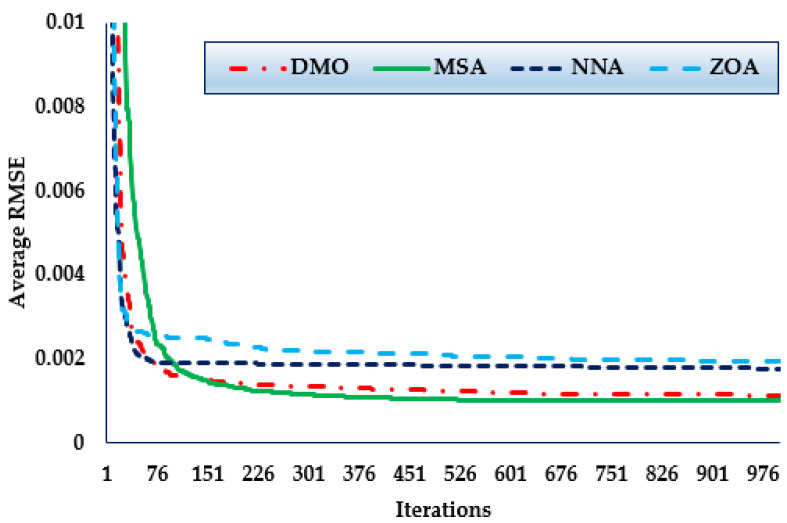
Mean convergence curves regarding MSA, NNA, DMO, and ZOA for Case 1.

Furthermore, the convergence trends in this scenario for the MSA, NNA, DMO, and ZOA approaches over the thirty simulated runs are depicted in Figure 5. As manifested in the figure, in terms of identifying lower RMSE objective values, the MSA technique has a higher rating than the others. It can also be noticed from the figure that the MSA method starts discovering undertaking areas after only 170 iterations, and then it realises the best solution. Comparing to the mean outcomes of the DMO, NNA, and ZOA techniques, the improvements of the MSA technique are 12.4%, 44.05%, and 48.88%, respectively, which support the superiority of the developed MSA for 1DM.

Table 3 compares the proposed MSA technique to several optimisation tools for the 1DM system that have been published in the scientific literature. The optimisers in the literature include GA with non-uniform mutation (NUM) [49], mutated BBO [50], teaching-learning-based optimisation (TLBO) [51], improved differential evolution (DE) [51], Chaotic PSO [51], artificial bee colonies (ABC) [52], harmony search-based algorithm (HSBA) [53], grey wolf optimiser (GWO) [54], JAYA optimiser [55], and comprehensive learning PSO [56]. As shown, the MSA technique surpasses other methods in terms of getting the smallest RMSE.

#### 4.1.2. Case 2: Application for 2DM System

The MSA, NNA, DMO, and ZOA techniques are employed to decrease the RMSE objective function for the 2DM model of commercial RTC France silicon PV cell. Table 4 displays the best outcomes for each algorithm for the seven 2DM parameters that were unknown during the experiment. The results demonstrate that MSA has excellent performance in contrast to its peers, which are newly developed techniques such as NNA, DMO, and ZOA. The table demonstrates that MSA earned the best RMSE value of 9.8271 × 10^−4^ from the comparison algorithms, which achieve RMSE values of 9.98712 × 10^−4^, 10.287 × 10^−4^, and 10.02 × 10^−4^ for NNA, DMO, and ZOA, respectively. Moreover, excellent improvements, according to these findings, have been obtained using the proposed MSA of 4.47%, 0.46%, and 1.89% with regard to the DMO, NNA, and ZOA techniques, respectively.

**Figure 6:**
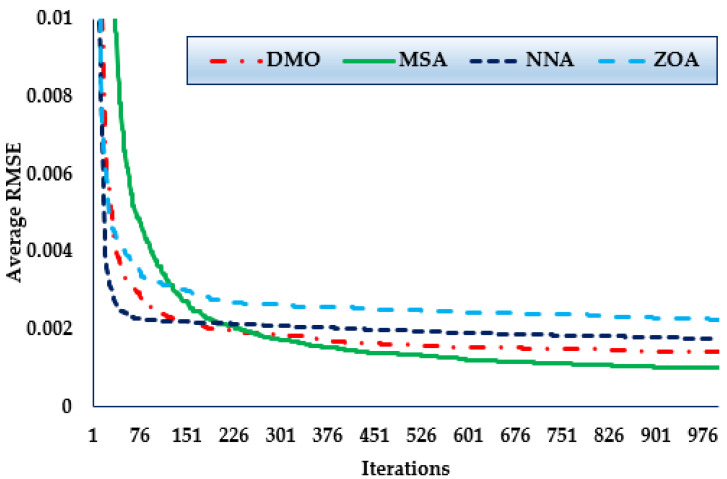
Mean convergence curves regarding MSA, NNA, DMO, and ZOA for Case 2.

Figure 6 shows the convergence trends for the MSA, NNA, DMO, and ZOA approaches in this scenario over the thirty simulated runs. The MSA technique outperforms the others in terms of identifying lower RMSE objective values. It can also be seen that the MSA method starts identifying undertaking areas after only 300 iterations and realises the best solution. Comparing to the mean outcomes of the DMO, NNA, and ZOA techniques, the improvements of MSA technique are 28.96%, 43.19%, and 55.81%, respectively, which support the superiority of the developed MSA for 1DM.

Table 5 compares the proposed MSA technique to several optimisation tools for the 2DM system that have been published in the scientific literature. The other optimisers are ABC [10], teaching–learning-based ABC [57], generalised oppositional TLBO [58], TLBO [59], the cat swarm algorithm [60], the sine–cosine approach [19], and the flower pollination algorithm [11]. As shown, the MSA technique surpasses other methods in terms of getting the smallest RMSE. Despite the BWOA [30] deriving a lower RMSE of 0.0009773823, it achieved impractical electrical parameters of the 2DM equivalent circuit. The reported ideality factor of the second diode was 2.4133546221726 [30], while the acceptable boundaries of the ideality factor are practically identified to be in the range [1,2].

#### 4.1.3. Case 3: Application for 3DM System

The MSA, NNA, DMO, and ZOA techniques are employed to decrease the RMSE objective function for the 3DM model of commercial RTC France silicon PV cell. Table 6 displays the best outcomes for each algorithm for the nine 3DM parameters that were unknown during the experiment. The results demonstrate that MSA has excellent performance, in contrast to its peers, which are newly developed techniques such as NNA, DMO, and ZOA. The table demonstrates that MSA earned the best RMSE value of 9.833 × 10^−4^ from the comparison algorithms, which achieve RMSE values of 10.05 × 10^−4^, 12.332 × 10^−4^ and 11.08 × 10^−4^ for NNA, DMO, and ZOA, respectively. Furthermore, outstanding improvements of 20.26%, 2.19%, and 11.29% were realised using the proposed MSA for the DMO, NNA, and ZOA procedures, respectively, according to these data.

**Figure 7:**
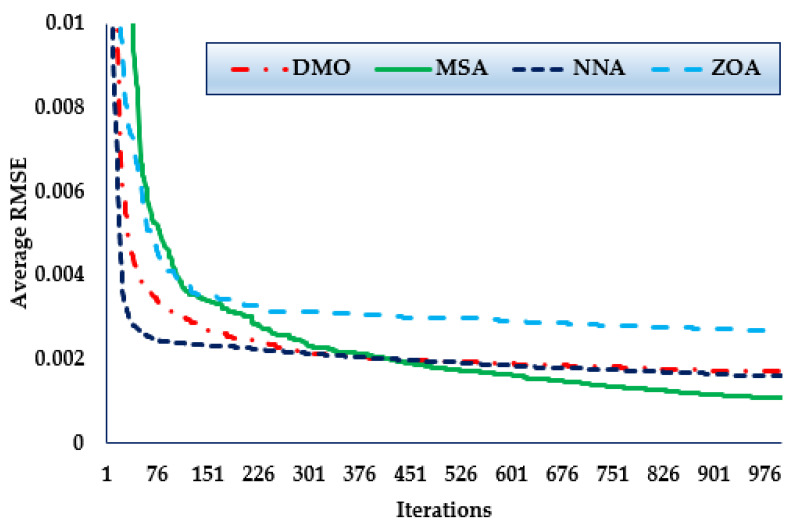
Mean convergence curves regarding MSA, NNA, DMO, and ZOA for Case 3.

Furthermore, the convergence trends in this scenario for the MSA, NNA, DMO, and ZOA approaches over the thirty simulated runs with a maximum of 1000 iterations, as depicted in Figure 7. As the figure shows, in terms of identifying lower RMSE objective values, the MSA technique has a higher rating than the others. It can be noticed also from the figure that the MSA method starts discovering undertaking areas after only 420 iterations, after which point it realises the best solution. Comparing to the mean outcomes of the DMO, NNA, and ZOA techniques, the improvements of MSA technique are of 37.71%, 32.71%, and 60.13%, respectively, which support the superiority of the developed MSA for 1DM.

**Figure 8:**
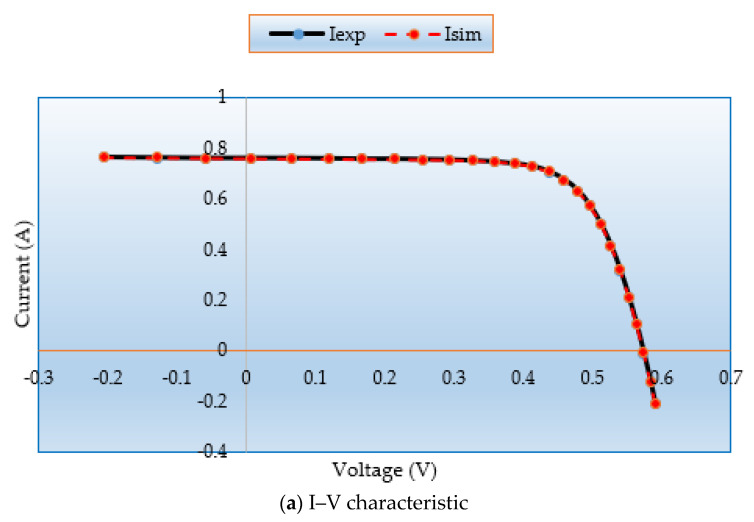

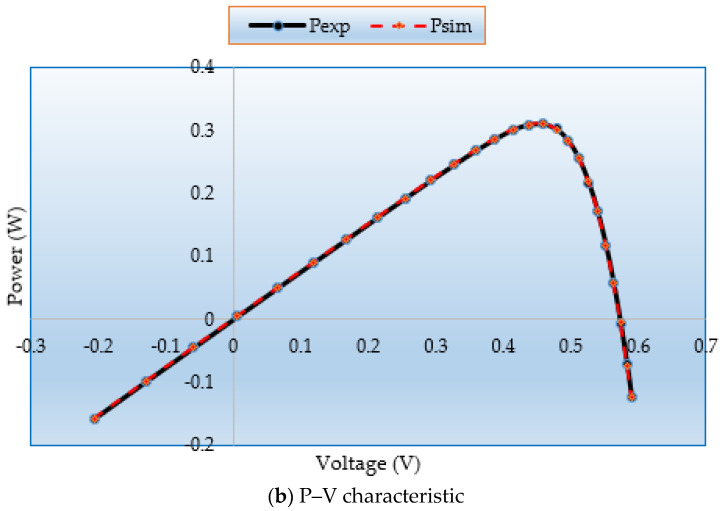
(**a**) I–V and (**b**) P–V curves regarding MSA for Case 3.

Figure 8a,b demonstrate the simulated and measured P-V and I-V characteristics for this model. It demonstrates that the MSA technique generated data are nearly identical to the experimental data, proving that the MSA technique successfully obtains the essential PV parameters.

#### 4.1.4. Statistical Assessment of MSA, NNA, DMO, and ZOA for Cases 1–3 (RTC France Cell)

**Figure 9:**
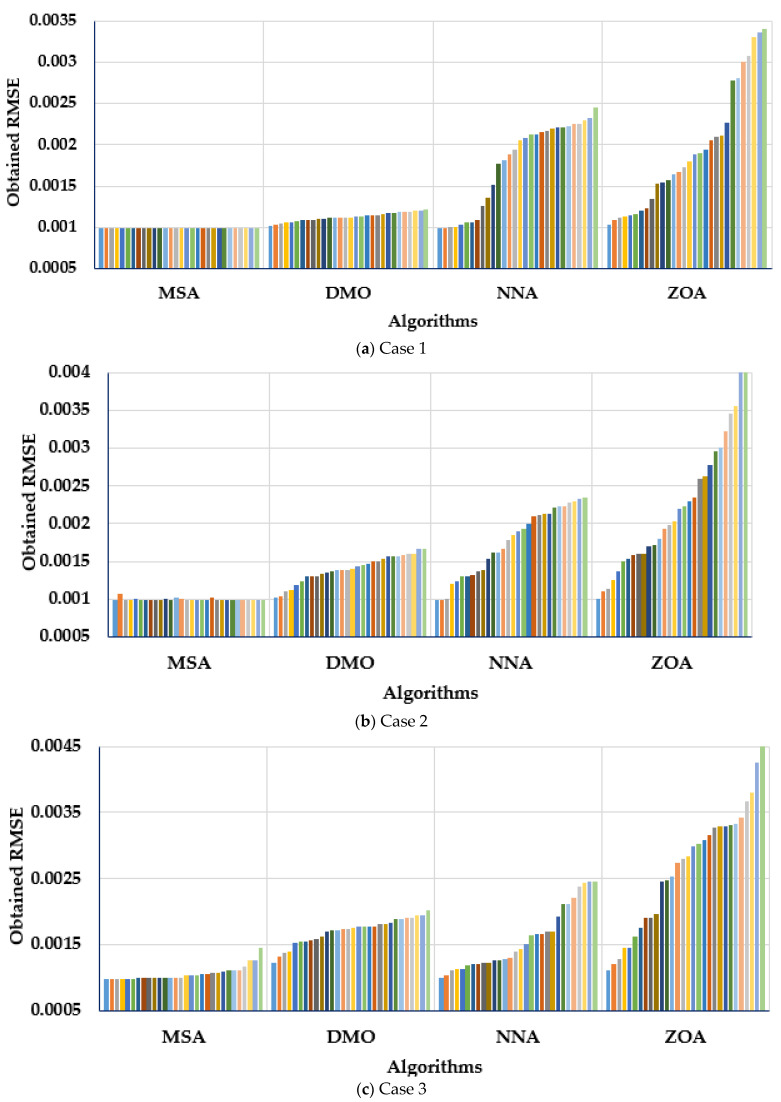
Obtained RMSE over the thirty separate applications regarding MSA, NNA, DMO, and ZOA for (**a**) Case 1, (**b**) Case 2 and (**c**) Case 3.

A statistical assessment of the RTC France cell’s MSA, NNA, DMO, and ZOA was made for the three cases studied. Figure 9 describes the obtained RMSE over the thirty separate applications regarding the MSA, NNA, DMO, and ZOA for Cases 1–3. Figure 9 depicts the values of the RMSE (objective score) related to each separate run. Therefore, each bar colour under each algorithm stands for each objective score that is obtained by this algorithm. Thus, the bars which are distinguished with lower lengths indicate the lower values of the obtained objective regarding the applied algorithm, which is denoted by the developed MSA for the three considered models.

As demonstrated, the MSA technique derives the maximum robustness, as it acquires the smallest RMSE with smallest standard deviation. For the first case, the presented MSA technique achieves the smallest standard deviation of 4.21 × 10^−12^, while the DMO, NNA, and ZOA techniques obtain counterparts of 5.42 × 10^−5^, 5.24 × 10^−4^, and 7.5 × 10^−4^, respectively. For Case 2, the demonstrated MSA approach obtains a standard deviation of 1.721 × 10^−5^, whereas the DMO, NNA, and ZOA procedures reach 1.8 × 10^−4^, 4.5 × 10^−4^, and 9.53 × 10^−4^, respectively. Thus, on the basis of standard deviation, the provided MSA approach outperforms the DMO, NNA, and ZOA procedures by 90.43%, 96.17%, and 98.19%, respectively. In Case 3, the provided MSA approach generates a standard deviation of 1.06 × 10^−4^, whereas the DMO, NNA, and ZOA procedures reach equivalents of 1.97 × 10^−4^, 4.69 × 10^−4^, and 9.26 × 10^−4^, respectively. Therefore, on the basis of standard deviation, the provided MSA approach outperforms the DMO, NNA, and ZOA procedures by 46.03%, 77.1357%, and 88.53%, respectively.

### 4.2. Second Test Investigation: Ultra 85-P PV Panel

#### 4.2.1. Case 4: Application for 1DM System

For Case 4, the MSA, NNA, DMO, and ZOA techniques are employed to decrease the RMSE objective function for the 1DM model of the commercial Ultra 85-P PV panel. Table 7 displays the best outcomes for each algorithm for the five 1DM parameters that were unknown during the experiment. The results demonstrate that MSA has excellent performance in contrast to its peers, which are newly developed techniques such as NNA, DMO, and ZOA. The table demonstrates that MSA earned the best RMSE value of 0.003563198 from the comparison algorithms, which achieve RMSE values of 0.003563424, 0.008172571, and 0.013722439 for NNA, DMO, and ZOA, respectively. Moreover, excellent improvements, according to the these finding, have been obtained using the proposed MSA of 56.4%, 0.01%, and 74.03% with regard to the DMO, NNA, and ZOA techniques, respectively.

**Figure 10:**
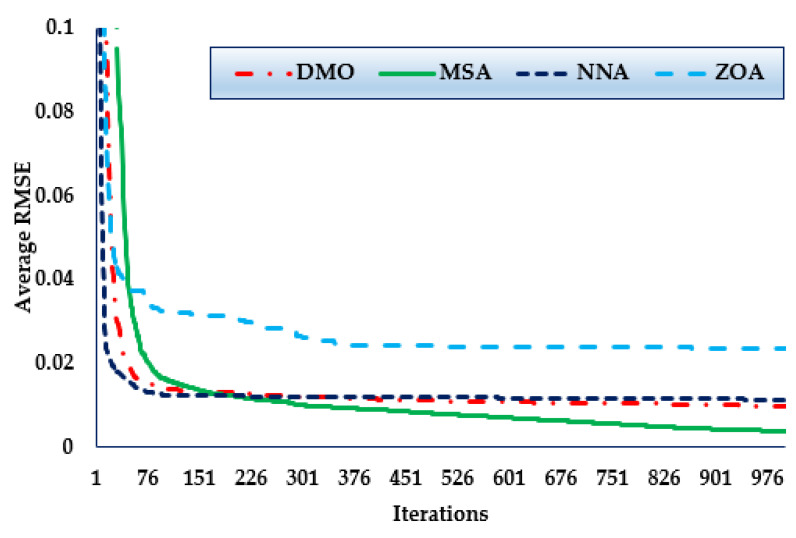
Mean convergence curves regarding MSA, NNA, DMO, and ZOA for Case 4.

Furthermore, the convergence trends in this scenario for the MSA, NNA, DMO, and ZOA approaches over the thirty simulated runs with a maximum of 1000 iterations, as depicted in Figure 10. As shown in the figure, in terms of identifying lower RMSE objective values, the MSA technique has a higher rating than the others. The figure also shows that the MSA method starts discovering undertaking areas after only 228 iterations, after which point it realises the best solution.

#### 4.2.2. Case 5: Application for 2DM System

The MSA, NNA, DMO, and ZOA techniques for Case 5 are employed to decrease the RMSE objective function for the 1DM model of commercial Ultra 85-P PV panel. Table 8 displays the best outcomes for each algorithm for the seven 2DM parameters that were unknown during the experiment. The results demonstrate that MSA has excellent performance in contrast to its peers, which are newly developed techniques such as NNA, DMO, and ZOA. The table demonstrates that MSA earned the best RMSE value of 0.003621422 from the comparison algorithms, which achieve RMSE values of 0.011392079, 0.004672962, and 0.013473226 for NNA, DMO, and ZOA, respectively. Moreover, excellent improvements, according to these findings, have been obtained using the proposed MSA of 68.21%, 22.5%, and 73.12% with regard to the DMO, NNA, and ZOA techniques, respectively.

**Figure 11:**
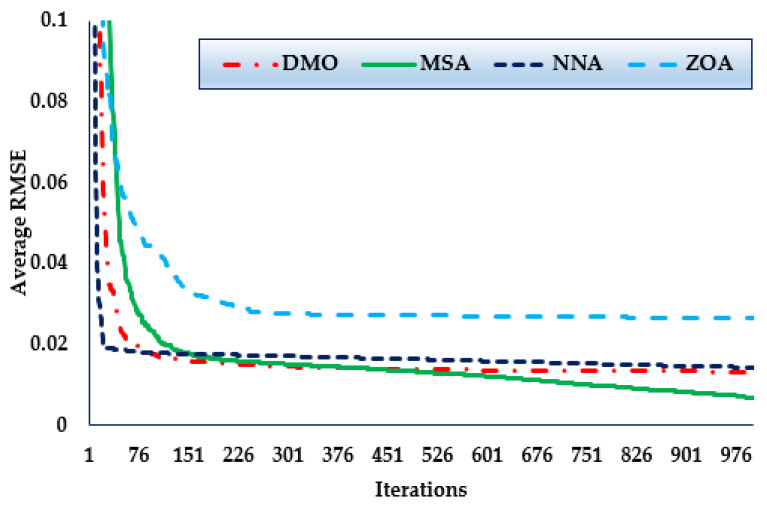
Mean convergence curves regarding MSA, NNA, DMO, and ZOA for Case 5.

Furthermore, the convergence trends in this scenario for the MSA, NNA, DMO, and ZOA approaches over the thirty simulated runs with a maximum of 1000 iterations, as depicted in Figure 11. The figures shows that, in terms of identifying lower RMSE objective values, the MSA technique has a higher rating than the others. It also shows that the MSA method starts discovering undertaking areas after only 450 iterations, after which point it realises the best solution.

#### 4.2.3. Case 6: Application for 3DM System

The MSA, NNA, DMO, and ZOA techniques, for Case 6 are employed to decrease the RMSE objective function for the 3DM model of commercial Ultra 85-P PV panel. Table 9 displays the best outcomes for each algorithm for the nine 2DM parameters that were unknown during the experiment. The results demonstrate that MSA has excellent performance in contrast to its peers, which are newly developed techniques such as NNA, DMO, and ZOA. The table demonstrates that MSA earned the best RMSE value of 0.005391459 from the comparison algorithms, which achieve RMSE values of 0.012520036, 0.007974089, and 0.012354068 for NNA, DMO, and ZOA, respectively. Moreover, excellent improvements, according to the these findings, have been obtained using the proposed MSA of 56.94%, 32.39%, and 56.36% with regard to the DMO, NNA, and ZOA techniques, respectively.

**Figure 12:**
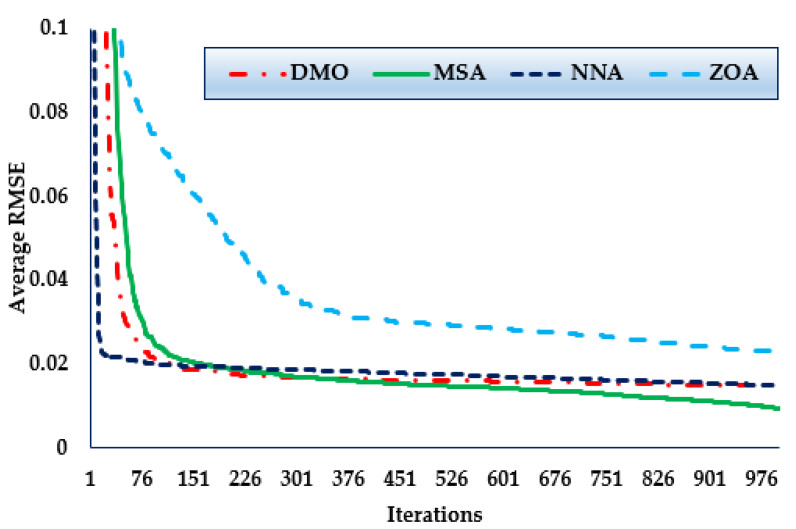
Mean convergence curves regarding MSA, NNA, DMO, and ZOA for Case 6.

Furthermore, the convergence trends in this scenario for the MSA, NNA, DMO, and ZOA approaches over thirty simulated runs with a maximum of 1000 iterations, as depicted in Figure 12. As shown in the figure, in terms of identifying lower RMSE objective values, the MSA technique has a higher rating than the others. It also shows that the MSA method starts discovering undertaking areas after only 300 iterations, after which point it realises the best solution.

**Figure 13:**
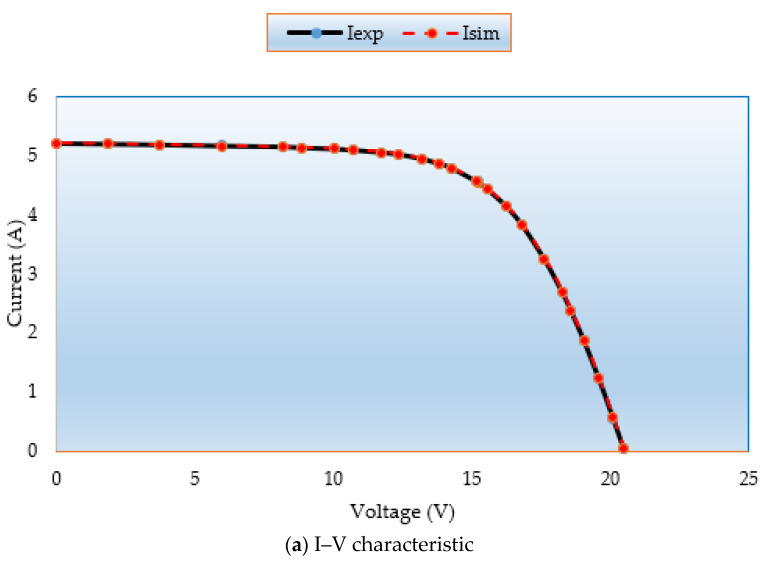

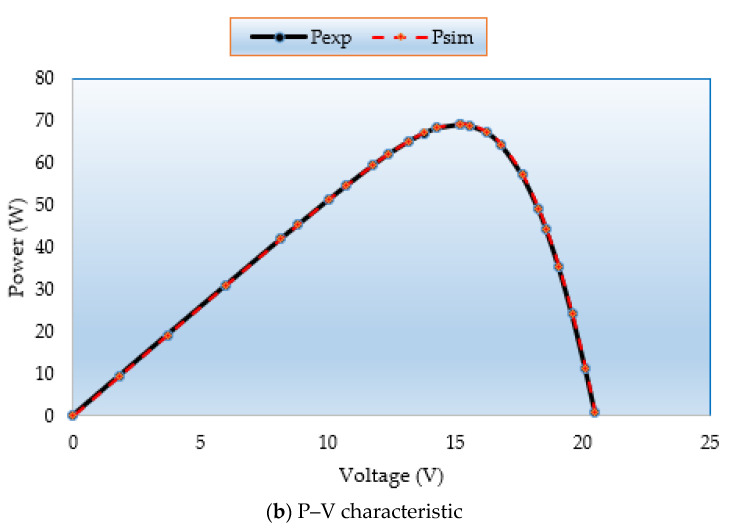
(**a**) I–V and (**b**) P–V curves regarding MSA for Case 6.

Figure 13a,b demonstrate the simulated and measured P–V and I–V characteristics for this model. It demonstrates that the MSA technique generated data are nearly identical to the experimental data, proving that the MSA technique successfully obtains the essential PV parameters.

#### 4.2.4. Statistical Assessment of MSA, NNA, DMO, and ZOA for Cases 4–6 (Ultra 85-P PV Panel)

**Figure 14:**
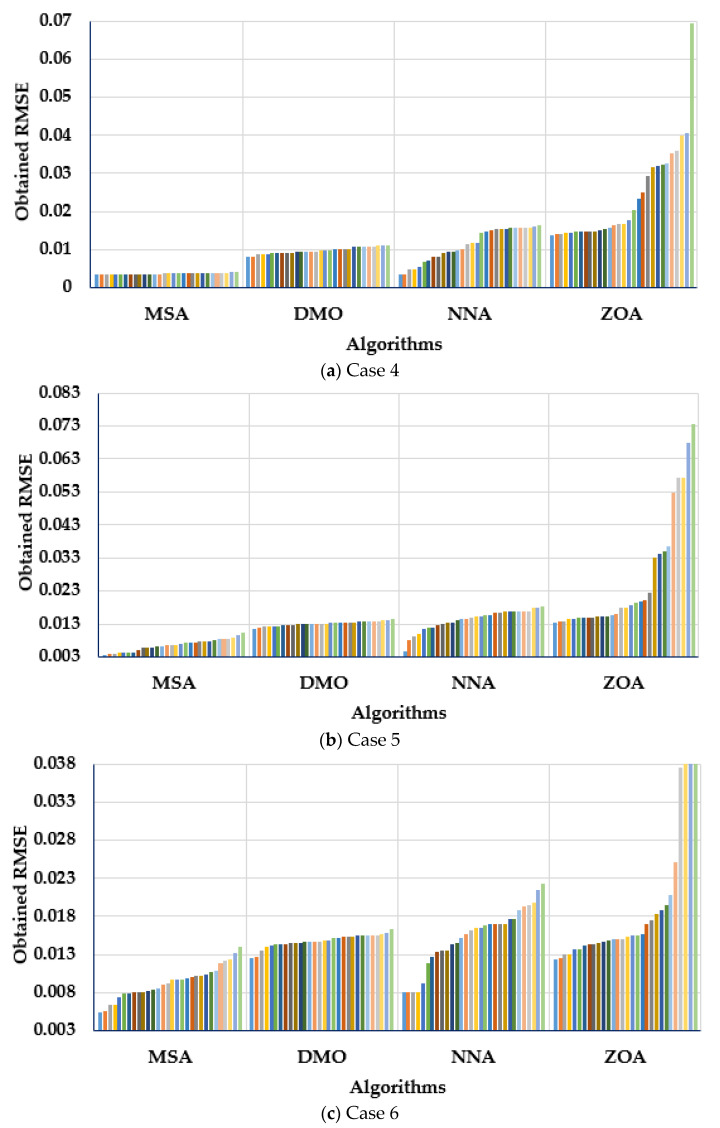
Obtained RMSE over the thirty applications regarding MSA, NNA, DMO, and ZOA for (**a**) Case 4, (**b**) Case 5 and (**c**) Case 6.

A statistical assessment of the Ultra 85-P PV panel’s MSA, NNA, DMO, and ZOA for the Cases 4–6 was made. Figure 14 describes the obtained RMSE over the thirty separate applications regarding MSA, NNA, DMO, and ZOA for Cases 4–6. As demonstrated, the MSA technique derives the maximum robustness as it acquires the smallest mean value of the RMSE over the thirty separate applications. For Case 4, the presented MSA technique achieves the smallest mean RMSE of 0.003702, while the DMO, NNA, and ZOA techniques obtain counterparts of 0.009755, 0.011266, and 0.023513, respectively. The provided MSA approach outperforms the DMO, NNA, and ZOA procedures by 62.05%, 67.14%, and 84.25%, respectively. For Case 5, the demonstrated MSA approach obtains the smallest mean RMSE of 0.006614, whereas the DMO, NNA, and ZOA procedures reach 0.013098, 0.014244, and 0.026398, respectively. The provided MSA approach outperforms the DMO, NNA, and ZOA procedures by 49.05%, 53.57%, and 74.95%, respectively. In Case 6, the provided MSA approach generates the least mean RMSE of 0.009312, whereas the DMO, NNA, and ZOA procedures reach equivalents of 0.014788, 0.014874, and 0.023032, respectively. The provided MSA approach outperforms the DMO, NNA, and ZOA procedures by 37.03%, 37.4%, and 59.57%, respectively.

The time complexity of MSA refers to the computational resources required by the algorithm to reach a solution. This complexity can be influenced by several factors, such as the problem size, the number of iterations or generations, and the complexity of the fitness evaluation function. Analysing the time complexity of MSA involves understanding the number of fitness evaluations needed and how it scales with problem size, which is tabulated for each model in Table 10. As shown, the complexity is increased by 40% when the considered model is changed from the 1DM to the 2DM, and it is increased by 28.57% when the considered model is changed from the 2DM to the 3DM.

## 5. Conclusions

This study constructs a novel nature-inspired metaheuristic algorithm of MSA technique inspired by the hunting techniques of praying mantises and uses it to extract the parameters of electrical 1DM, 2DM, and 3DM systems. The created MSA employs three optimisation operators to represent exploration, exploitation, improving exploitation, and exploration, namely hunting for prey, assaulting prey, and sexual cannibalism. In this context, two commercial solar PV systems are handled: the RTC France PV cell and the Shell PowerMax Ultra 85-P PV panel. The performance of the method is compared to newly built optimisers of neural network optimisation algorithm (NNA), dwarf mongoose optimisation algorithm (DMO), and zebra optimisation algorithm (ZOA). The robustness analysis is executed by performing the MSA, NNA, DMO, and ZOA techniques for thirty separate runs, and the indices of the best, mean, worst, and standard deviation are tabulated for the three investigated models for all PV modules under study. The proposed MSA method is intended to optimise the parameters for optimal value of RMSE accomplishments. In comparison, the suggested MSA approach outperforms them in terms of superiority and statistical robustness, with greater improvement percentages. The accurate solution of PV cell/panel models provides additional support that the proposed MSA outperforms recently reported optimiser tools in the literature. We recommend the variations in input parameters, noise in the data, or uncertainties in the PV module characteristic are incorporated in future work. The efficient application of the MSA in this study can also be extended to several other power system engineering problems, such as economic dispatch, combined heat and power optimisation [61,62,63], and the integration of renewable sources [64], etc.

## Figures and Tables

**Table 1:** The margins range for the cell parameters.

Parameter	RTC France PV Cell		Ultra 85-P PV Panel	
	Lower	Upper	Lower	Upper
*I_s_*_1_, *I_s_*_2_, *I_s_*_3_ (μA)	0.00	1.00	0.00	10.00
*I_Ph_* (A)	0.00	1.00	0.00	10.00
*R_sh_* (Ω)	0.00	100.00	0.00	100.00
*R_s_* (Ω)	0.00	0.50	0.00	2.00
*η*1, *η*2, *η*3 per cell	1.00	2.00	1.00	2.00

**Table 2:** Electrical parameters attained by MSA, NNA, DMO, and ZOA for Case 1.

Applied Technique	MSA	DMO	NNA	ZOA
*I_Ph_* (*A*)	0.7607755	0.7605558	0.7607653	0.7606034
*R_sh_* (Ω)	0.0363771	0.0358427	0.0362887	0.0357996
*R_s_* (Ω)	53.7185260	58.9141185	54.3393156	60.0352518
*I_s_*_1_ (*A*)	0.0000003	0.0000004	0.0000003	0.0000004
*η*1	1.4811836	1.4943328	1.4834317	1.4966056
RMSE	0.0009860	0.0010212	0.0009869	0.0010309
Difference compared to MSA	-	3.52 × 10^−5^	9.15 × 10^−7^	4.48 × 10^−5^
Improvement	-	3.45%	0.09%	4.35%

**Table 3:** Comparative assessment regarding MSA, NNA, DMO, and ZOA for Case 1.

Algorithms	RMSE
MSA	0.0009860
DMO	0.0010212
NNA	0.0009869
ZOA	0.0010309
GA with NUM [49]	9.8618 × 10^−4^
Mutated BBO [50]	9.8634 × 10^−4^
TLBO [51]	9.8733 × 10^−4^
ABC [52]	10 × 10^−4^
Improved DE [51]	9.89 × 10^−4^
Chaotic PSO [51]	13.8607 × 10^−4^
HSBA [53]	9.95146 × 10^−4^
GWO [54]	75.011 × 10^−4^
JAYA [55]	9.8946 × 10^−4^
Comprehensive Learning PSO [56]	9.9633 × 10^−4^

**Table 4:** Electrical parameters attained by MSA, NNA, DMO, and ZOA for Case 2.

Applied Technique	MSA	DMO	NNA	ZOA
*I_Ph_* (A)	0.76078221	0.761086003	0.760790742	0.76087427
*R_sh_* (Ω)	0.03665767	0.036452844	0.036596751	0.036829377
*R_s_* (Ω)	55.12971603	56.0407128	53.33855515	50.98794622
*I_s_*_1_ (A)	2.43012 × 10^−7^	3.81141 × 10^−7^	1.84217 × 10^−7^	2.56794 × 10^−7^
*η*1	1.457158843	1.83357911	1.448493654	1.461518645
*I_s_*_2_ (A)	6.04558 × 10^−7^	2.38858 × 10^−7^	1.80839 × 10^−7^	1.16674 × 10^−7^
*η*2	1.996965209	1.458364626	1.589535404	1.777597367
RMSE	0.000982718	0.001028696	0.000987219	0.001001673
Difference compared to MSA	-	4.60 × 10^−5^	4.50 × 10^−6^	1.90 × 10^−5^
Improvement	-	4.47%	0.46%	1.89%

**Table 5:** Comparative assessment regarding MSA, NNA, DMO, and ZOA for Case 2.

Algorithms	RMSE
MSA	0.000982718
DMO	0.001028696
NNA	0.000987219
ZOA	0.001001673
BWOA [30] *	0.0009773823 *
ABC	1.28482 × 10^−3^
Teaching–learning–based ABC	1.50482 × 10^−3^
Generalised oppositional TLBO	4.43212 × 10^−3^
TLBO	1.52057 × 10^−3^
Cat swarm algorithm	1.22 × 10^−3^
Sine cosine approach	9.86863 × 10^−4^
Comprehensive learning PSO	1.3991 × 10^−3^
Flower pollination algorithm	1.934336 × 10^−3^

* indicates impractical solution.

**Table 6:** Electrical parameters attained by MSA, NNA, DMO, and ZOA for Case 3.

Applied Technique	MSA	DMO	NNA	ZOA
*I_Ph_* (A)	0.760771771	0.760598554	0.760746273	0.760933659
*R_sh_* (Ω)	0.036624048	0.035617495	0.037016425	0.035602637
*R_S_* (Ω)	54.85092436	71.78477566	58.21689511	56.95493745
*I_S_*_1_ (A)	2.47337 × 10^−7^	4.56677 × 10^−7^	5.28442 × 10^−7^	1.54528 × 10^−7^
*η*1	1.458909504	1.862177339	1.585110202	1.444120881
*I_S_*_2_ (A)	1.8128 × 10^−7^	4.77356 × 10^−7^	1.34494 × 10^−8^	4.93567 × 10^−8^
*η*2	1.982050848	1.685843012	1.289423782	1.699258208
*I_S_*_3_ (A)	2.8619 × 10^−7^	1.05595 × 10^−7^	1.01943 × 10^−7^	3.50553 × 10^−7^
*η*3	1.933021037	1.410321073	1.999680734	1.631958875
RMSE	0.000983323	0.001233216	0.001005292	0.001108423
Difference compared to MSA	-	2.50 × 10^−4^	2.20 × 10^−5^	1.25 × 10^−4^
Improvement	-	20.26%	2.19%	11.29%

**Table 7:** Electrical parameters attained by MSA, NNA, DMO, and ZOA for Case 4.

Applied Technique	MSA	DMO	NNA	ZOA
*I_Ph_* (A)	5.227492636	5.209587967	5.227413736	5.180799436
*R_sh_* (Ω)	0.011074354	0.010647702	0.011071209	0.010118476
*R_s_* (Ω)	3.764442466	4.952758368	3.770662178	23.47359277
*I_s_*_1_ (A)	1.01117 × 10^−5^	1.64942 × 10^−5^	1.01497 × 10^−5^	3.1682 × 10^−5^
*η*1	1.56462094	1.624721679	1.565062489	1.711091626
RMSE	0.003563198	0.008172571	0.003563424	0.013722439
Difference compared to MSA	-	0.004609373	2.26221 × 10^−7^	0.010159241
Improvement	-	56.40%	0.01%	74.03%

**Table 8:** Electrical parameters attained by MSA, NNA, DMO, and ZOA for Case5.

Applied Technique	MSA	DMO	NNA	ZOA
*I_Ph_* (A)	5.225245297	5.192576302	5.217578751	5.17870078
*R_sh_* (Ω)	0.011028319	0.01018628	0.010966592	0.010245894
*R_s_* (Ω)	3.894603375	10.09141719	4.80950381	23.72714823
*I_s_*_1_ (A)	1.84526 × 10^−7^	4.1711 × 10^−6^	2.87875 × 10^−5^	1.87291 × 10^−5^
*η*1	1.995046933	1.602714205	2	1.716772886
*I_s_*_2_ (A)	1.06059 × 10^−5^	2.46318 × 10^−5^	5.64295 × 10^−6^	1.01294 × 10^−5^
*η*2	1.57035711	1.726106004	1.517105615	1.669564389
RMSE	0.003621422	0.011392079	0.004672962	0.013473226
Difference compared to MSA	-	0.007770657	0.00105154	0.009851804
Improvement	-	68.21%	22.50%	73.12%

**Table 9:** Electrical parameters attained by MSA, NNA, DMO, and ZOA for Case 6.

Applied Technique	MSA	DMO	NNA	ZOA
*I_Ph_* (A)	5.211524612	5.20406249	5.207590683	5.206605809
*R_sh_* (Ω)	0.010830847	0.010216306	0.010615902	0.010011148
*R_s_* (Ω)	5.096501608	9.728685738	6.482032244	7.526568705
*I_s_*_1_ (A)	4.95291 × 10^−6^	1.46832 × 10^−5^	2.02445 × 10^−11^	5.96796 × 10^−6^
*η*1	1.592291062	1.639062307	1.945438395	1.845134969
*I_s_*_2_ (*A*)	7.29486 × 10^−6^	6.86358 × 10^−6^	5.17631 × 10^−6^	1.67964 × 10^−5^
*η*2	1.590393766	1.862699049	1.530454645	1.774759034
*I_s_*_3_ (A)	5.29217 × 10^−6^	2.044 × 10^−6^	3.01439 × 10^−6^	1.17361 × 10^−6^
*η*3	1.984877988	1.973364739	1.882800822	1.651100572
RMSE	0.005391459	0.012520036	0.007974089	0.012354068
Difference compared to MSA	-	0.007128577	0.00258263	0.006962609
Improvement	-	56.94%	32.39%	56.36%

**Table 10:** Time complexity of MSA in solving each PV model extraction problem.

	1DM	2DM	3DM
No of solutions	100	100	100
No of iterations	1000	1000	1000
Dim	5	7	9
Complexity using O notation	O(500,000) × O(F(x)).	O(700,000) × O(F(x)).	O(900,000) × O(F(x)).

## Data Availability

Not applicable.

## Appendix A

The settings of the MSA are stated in Table A1 in the Appendix A.

**Table A1:** MSA settings in solving each PV model extraction problem.

Value	Parameter Description
*p* = 0.5	A probability to exchange between the exploration and exploitation stages
A = 1.0	Length of the archive
a = 0.5	A probability of the strike’s failure
P = 2	A recycling factor to exchange between pursuers and spearers
alp = 6	The gravitational acceleration rate of the mantis’s strike
Pc = 0.2	The percentage of sexual cannibalism
